# Numerical Simulation of Impregnation Process of Reactive Injection Pultrusion for Glass Fiber/PA6 Composites

**DOI:** 10.3390/polym14040666

**Published:** 2022-02-10

**Authors:** Xueliang Ding, Quanguo He, Qun Yang, Suwei Wang, Ke Chen

**Affiliations:** 1 Chongqing Copolyforce New Materials Co., Ltd., Chongqing 401332, China; dingxxueliang@163.com (X.D.); hequanguo@vip.sina.com (Q.H.); 2 Chongqing Research Institute Co., Ltd. of China Coal Technology & Engineering Group Corporation, Chongqing 400039, China; 3 College of Mechanical and Electrical Engineering, Beijing University of Chemical Technology, Beijing 100029, China; honor0612@163.com; 4 National Special Superfine Powder Engineering Research Center of China, School of Chemistry and Chemical Engineering, Nanjing University of Science and Technology, Nanjing 210094, China

**Keywords:** thermoplastic composites, reactive injection pultrusion, impregnation, numerical simulation

## Abstract

Pultrusion of thermoplastic composites has been the hotspot of manufacturing high-performance thermoplastic composites in recent years. The optimization of process parameters in the pultrusion usually needed repeated attempts, which wasted lots of manpower and material resources. A numerical simulation method can accelerate the optimization of process parameters. In this work, the impregnation process of reactive injection pultrusion for glass fiber reinforced nylon 6 (GF/PA6) composites was modeled and numerically simulated by a finite element/controlled volume (Fe/CV) method. Based on Darcy’s law, the impregnation process can be regarded as the two-phase flow (liquid resin and air) in porous media (undirectional glass fibers). The distribution of resin flow during the impregnation was explored. The effects of pulling rate and injection pressure on the impregnation time and resin reflux distance were analyzed, and the appropriate range of relevant process parameters was determined. The results showed that increasing the pulling rate can significantly control the reflux distance of resin in the impregnation mold and shorten the impregnation time, but too high a pulling rate would increase the impregnation time. Increasing the injection pressure can greatly shorten the resin impregnation time, but it would significantly increase the resin reflux distance. This work can effectively guide the subsequent optimization of process parameters of reactive injection pultrusion for GF/PA6 composites.

## 1. Introduction

Pultrusion is a continuous process of manufacturing composite profiles by impregnating continuous reinforcement fibers or fabrics guided into a die to attain the desired cross-sectional profile followed by heating to achieve curing of the material [1,2]. It has the advantages of controllable fiber content, high raw material utilization, and high production efficiency. The process allows the manufacture of pultruded profiles with virtually unlimited length and higher flexibility and tensile strength compared to those prepared with any other reactive polymer process [3,4]. Pultruded products have the advantages of light weight, high strength, and high corrosion resistance and are widely used in construction, transportation, automotive manufacturing, aircraft, power transmission, and other fields [5,6,7]. For example, the windshield bracket of the latest BMW I3 is the pultruded continuous carbon fiber reinforced PA6 composites product, which can reduce the mass of the whole vehicle.

During the pultrusion, the impregnation of resin to fiber reinforcement is the key to determine the properties of the final product. The traditional open-bath impregnation method can achieve good impregnation, but the volatile organic compounds (VOC) produced may involve health risks [8]. Closed-injection impregnation was an efficient impregnation strategy in recent years. Once launched, it was favored by many pultrusion manufacturers and was used in the pultrusion of epoxy-based, polyurethane-based composites [9,10]. With closed-injection impregnation technology, high-speed pultrusion can be achieved by optimizing the pulling speed, injection pressure, and structural parameters of the impregnation box, and the maximum production speed can reach 3 m/min by KraussMaffei’s iPul technology at K2019 exhibition [11]. Pultrusion involves numerous variables that affect the quality and mechanical properties of the final products [12]. Improper pulling rate or excessive low die temperature would lead to the decline of product performance and even the failure of production. Traditional “trial and error” method consumed too much manpower and material costs, when optimizing the pultrusion process parameters. In recent years, with the rapid development of computer technology, modeling of pultrusion has attracted more and more researchers’ interest [13,14,15]. Numerical simulation can effectively shorten the time of identifying appropriate process parameter. Especially for the impregnation process of closed injection impregnation, it is the most significant target of pultrusion modeling.

Kim et al. [16] were the first to carry out research about impregnation modeling and established a one-dimensional permeability model based on Darcy’s law. Kommu et al. [17] used the finite element/control volume (Fe/CV) method to solve the resin flow equation in the two-dimensional calculation domain. Then, Rahatekar et al. [18] also established a two-dimensional resin flow model in the impregnation box. It was found that, in order to ensure the complete impregnation of fiber reinforcement, the injection pressure should be increased when increasing the pultrusion speed, raising fiber volume fraction, or reducing the compression ratio. Srinivasagupta et al. [19] found that the structure parameters of impregnation box had significant influence on the injection pressure required to penetrate the fiber. Liu et al. [20,21] reached the same conclusion when using the finite element/nodal volume (Fe/NV) method to simulate the transient modeling of resin flow front. Jeswani et al. [22] used Fe/CV method to establish a three-dimensional model of resin flow impregnated fiber reinforcement during injection pultrusion, and predicted the flow front of resin in the impregnation box. Masuram et al. [23,24] and Roux et al. [25,26] further considered the influence of compression of fiber reinforcement on resin flow and impregnation effect, so that the numerical simulation of impregnation process of injection pultrusion molding was closer to the reality. These works mainly focused on thermosetting composite systems.

This current work focused on the modeling and numerical simulation of impregnation 3D flow process during thermoplastic injection pultrusion of glass fiber reinforced nylon 6 (GF/PA6) composites. This technology combined PA6 anionic polymerization and injection pultrusion. The infiltration flow of resin to continuous reinforcement during impregnation was numerically simulated by ANSYS CFX 15.0 software. The effects of process parameters such as pultrusion speed and injection pressure on the distribution of resin flow front and backflow distance were analyzed, which provided a theoretical basis for optimizing process parameters of pultrusion.

## 2. Materials and Methods

### 2.1. Materials

The pultruded profile with section size of 20 × 4 mm^2^ was investigated in this work. The undirectional reinforcements were E-glass roving (ECT 4301R, 2400 tex, *D*_f_ = 17 μm), provided by Chongqing International Composite Materials Co., Ltd., Chongqing, China. Caprolactam (PA6 monomer) was the resin for impregnating reinforcements, which was purchased from BASF Co., Ltd., Shanghai, China. The initiators C10 (2 mol/kg) and activators C20 (2 mol/kg) were kindly provided by Sinopharm Chemical Reagent Co., Ltd., Shanghai, China. A C20 concentration of 1.0 mol% and a C10 concentration of 2.0 mol% in caprolactam were optimized by our previous work [27,28] to be optimal formulations for pultrusion.

### 2.2. Impregnation of Thermoplastic Injection Pultrusion

**Figure 1:**
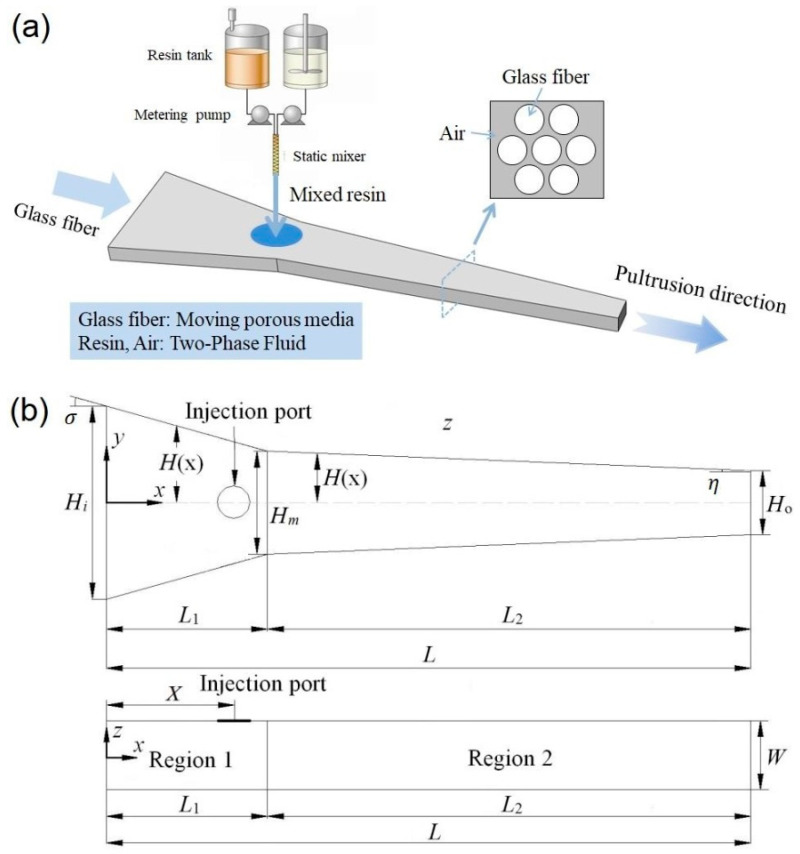
(**a**) Schematic diagram of injection impregnation process; (**b**) geometric parameters of impregnation box cavity.

During the injection pultrusion, the impregnation of unidirectional reinforcement took place in the impregnation box, as showed in Figure 1a. Continuous unidirectional fiber bundles can be regarded as the moving porous media during the pultrusion process. At the initial stage, the pores of the porous media were filled with hot air. The mixed two-component resins were injected into the closed impregnation box, and penetration of the fiber bundles was implemented by the metering pumps of reaction injection molding (RIM) device. This physical process can be regarded as the two-phase transient flow of hot air and liquid resin in moving porous media.

If the impregnation box was taken as the research object, the coordinate axis was set at the center of the fiber entrance, as shown in Figure 1b. According to the different cone angles, the calculation domain is divided into Region 1 and Region 2. The specific geometric parameters are shown in Table 1.

The heating temperature of the impregnation box was set as 100 ℃; under this condition, the physical parameters of air and liquid resin are present in Table 2.

### 2.3. Modeling of Injection Pultrusion

In order to establish a mathematical model of the pultrusion impregnation process, the following assumptions are made:

(1) Low viscosity resin is incompressible Newtonian fluid;
(2) The flow of resin through the fiber reinforcement (porous medium) complied with Darcy’s law [13];
(3) The impregnation process was isothermal, and the viscosity of the resin system was constant;
(4) The initial pressure was atmospheric pressure (101.325 kPa);
(5) The influence of capillary force was neglected.

Darcy formula porous medium model was used to calculate the flow process of resin system in fiber reinforcement. There were gas-liquid two-phase flows (hot air and resin) in porous media; the two continuous phases were completely layered, and the interface was clear. Therefore, it can be assumed that the two continuous phases shared the same velocity field [29]. During the impregnation process, the gas–liquid two-phase flows maintained mass conservation and momentum conservation, so the continuity equation and momentum equation were as follows:

(1) $$\frac{\partial \left(\rho \right)}{\partial t}+\nabla \cdot \left(\rho \bar{U}\right)=0$$

(2) $$\frac{\partial \left(\rho \bar{U}\right)}{\partial t}+\nabla \cdot \left(\rho \cdot \bar{U}⊗\bar{U}\right)=-\nabla P\cdot \delta +\nabla \cdot \left(\eta \cdot \left(\nabla \bar{U}+{\left(\nabla \bar{U}\right)}^{T}\right)\right)-\frac{\eta }{K}\bar{U}+S$$

where ρ was the volume fraction weighted average density of liquid resin and air, *t* was the time, $\bar{U}$ was the apparent velocity, the physical velocity $U=\varphi \bar{U}$, δ was the unit vector, *P* was the impregnation pressure, *η* was the weighted average viscosity of volume fraction of liquid resin and air, and  K was the permeability (for unidirectional fiber, the permeability takes different values in different directions). *S* was the momentum source term generated by gravity. In this model, it can be expressed as:

(3) $$S=\left(\rho -\rho_{\mathrm{ref}}\right)\cdot g$$

where $\rho_{\mathrm{ref}}$ was the reference density.

The gas–liquid two-phase seepage flow between fiber bundle was laminar flow, and the volume fraction method was used to track the interface. The mass conservation equation of liquid resin phase was:

(4) $$\frac{\partial \left(\alpha_{r}\rho_{r}\right)}{\partial t}+\nabla \cdot \left(\alpha_{r}\rho_{r}\cdot U\right)=0$$

(5) $$\alpha_{r}+\alpha_{\mathrm{air}}=1$$

where the volume fraction of liquid resin was $\alpha_{r}$, the volume fraction of air was $\alpha_{\mathrm{air}}$, $\rho_{r}$ was the density of the liquid resin, $\rho_{\mathrm{air}}$ was the density of air, $\eta_{r}$ was the viscosity of the liquid resin, and $\eta_{\mathrm{air}}$ was the viscosity of air. Then,

(6) $$\rho =\alpha_{r}\rho_{r}+\left(1-\alpha_{r}\right)\rho_{\mathrm{air}}$$

(7) $$\alpha_{r}+\alpha_{\mathrm{air}}=1$$

The sum of the porosity φ of the porous medium and the fiber volume fraction $V_{f}$ was 1; thus,

(8) $$\varphi =1-V_{f}$$

For the conical impregnation box, the fiber volume fraction was not constant, but would gradually increase in the *X* direction according to the gradual reduction in the internal section of the box until the volume fraction $V_{f0}$ of the final product was reached at the outlet of the impregnation box. So, the fiber volume fraction $V_{f}\left(x\right)$ in the *X* direction can be expressed as:

(9) $$V_{f}\left(x\right)=V_{f0}\frac{H_{o}}{2H\left(x\right)}$$

In Equation (9), *H(x)* was a piecewise function, which was represented in Region 1 and Region 2 of the impregnation box, respectively:

(10) $$H\left(x\right)=\frac{\left(H_{m}-H_{i}\right)}{2L_{1}}x+\frac{H_{i}}{2},0\leq x\leq L_{1}$$

(11) $$H\left(x\right)=\frac{H_{0}-H_{m}}{2\left(L-L_{1}\right)}\left(x-L_{1}\right)+\frac{H_{m}}{2},L_{1}<x\leq L$$

In the pultrusion process of fiber-reinforced composites, permeability was considered to be the indicator of the difficulty of liquid resin flowing through fiber reinforcement. Pultruded products usually had high fiber content, and the permeability *K* was related to the structure of the fiber itself. This model adopts the permeability model proposed by Gebart [30]:

(12) $$K_{⊥}=C_{1}{\left(\sqrt{\frac{V_{\mathrm{fmax}}}{V_{f}}}-1\right)}^{2.5}R_{f}{}^{2}$$

(13) $$K_{∥}=\frac{8R_{f}^{2}}{c}\frac{{\left(1-V_{f}\right)}^{3}}{V_{f}^{2}}$$

where $K_{∥}$ was the permeability along the fiber axial direction (*X* direction), and $K_{⊥}$ was the permeability along the fiber transverse direction (*y* direction and *z* direction). The parameters are shown in Table 3.

**Figure 2:**
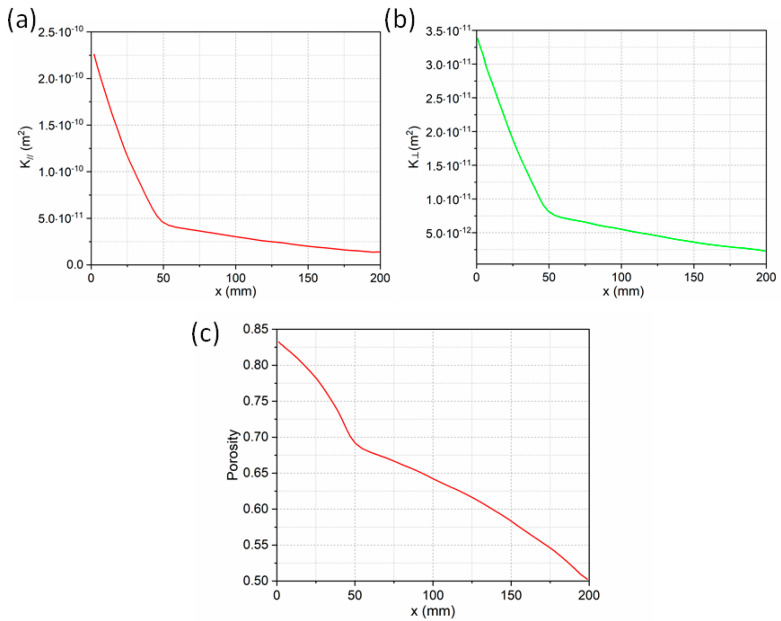
Relationship between fiber permeability, porosity, and *X*-axis coordinate: (**a**) permeability along fiber axis (*X*-axis direction); (**b**) permeability along the fiber radial (*Y*-axis direction); (**c**) porosity along the *X*-axis coordinate.

According to Equations (12) and (13) and the parameters in Table 3, when the volume fraction of fiber reinforcement in the final product was 50%, the relationship between the permeability (along the fiber axis and the fiber transverse direction) and the x coordinate is shown in Figure 2.

## 3. Simulation

### 3.1. Software Settings

**Figure 3:**
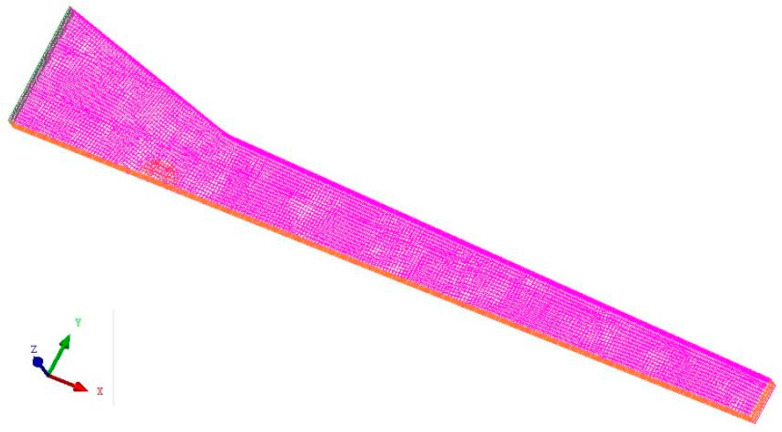
Mesh division of impregnation domain.

The geometric model of the simulation was drawn in Solidworks 2013 and meshed in ICEM CFD. The maximum size of the grid was set to 0.8 mm. The automatic volume mesh generation method was used to divide geometric model into more than 40,000 meshes dominated by hexahedral mesh, check the mesh quality, and delete the negative mass mesh, as shown in Figure 3.

The commercial finite element software ANSYS CFX 15.0 was used to simulate the process of resin infiltration into fiber in impregnation box, and the transient solution was carried out by the finite volume (FV) method. The time step was 0.05 s, and the total simulation time was 10.0 s. The unidirectional fiber area was set as the porous media area, and the buoyancy model was set according to the die inclination of 5°. The fluid phase adopted the homogeneous model, the flow was set as laminar flow, and there was no material transfer between the two continuous phases. The permeability of porous media domain was different along each direction, so it was set as an anisotropic model.

The solution mode adopted a high-order solution mode, which was accurate and reliable, and the transient solutions were set as implicit second-order backward Euler mode. The maximum number of iteration steps was set to 20 times, and the root mean square residual was 10^−4^, and the accuracy can meet the calculation requirements of this model [31]. Multiphase control adopted a volume fraction coupling method, and the flow front of resin in unidirectional reinforced fiber was tracked by volume fraction (VOF) method. This method defined a fluid volume function that was the ratio of the volume of the target fluid to the grid volume. As long as the value of this function on each grid was known in the flow field, the moving interface could be tracked.

### 3.2. Boundary Conditions

**Figure 4:**
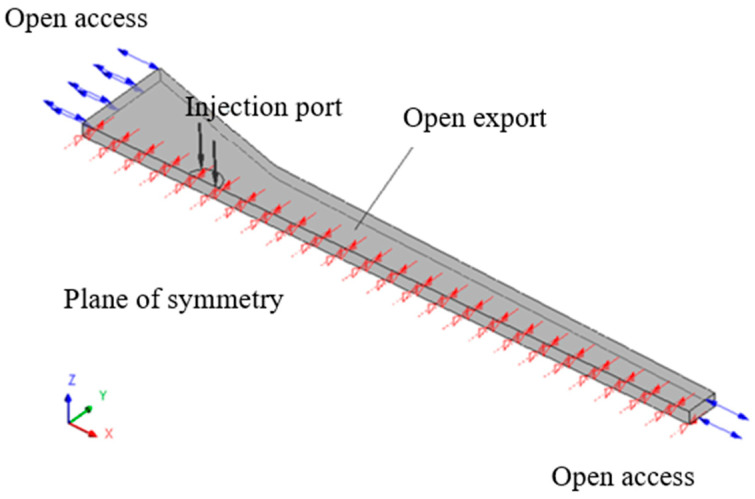
Initial and boundary conditions of impregnation process.

The impregnation box had axisymmetric geometric structure, and the resin injection port was located on the central axis. Therefore, in order to reduce the simulation computation work, analyzing half of the impregnation box can reflect the process of resin infiltration into the pores between the fiber in the entire impregnation box, and the schematic diagram is shown in Figure 4.

In the initial state, the reference pressure in the computational domain was atmospheric pressure $P_{\mathrm{atm}}$, the porous media were completely filled with air, the liquid phase volume fraction of resin $\alpha_{r}=0$, and the initial velocity of the air was 0. In this model, the impregnation box was convergent flow channel, the velocity in the *x*-axis direction of the porous media domain was $u_{0}$, that was, the pultrusion velocity of the composite. The velocity $V_{0}$ along the *y*-axis direction can be simplified by the cone angle and the pultrusion velocity in the *x*-axis direction, which is expressed as:

(14) $$v_{0}=-u_{0}\left(\frac{y}{H\left(x\right)}\right)tan\theta$$

where $0\leq X\leq L_{1}$, tanθ=tanσ, and $L_{1}\leq X\leq L_{2}$, tanσ=tanη.

The boundary conditions were as follows:

(1) Injection port: the injection port was the pressure inlet, the injection pressure was $P_{i}$, and the volume fraction of resin was $\alpha_{r}=1$;
(2) Front and rear outlet of impregnation box: at the front and rear channels of the impregnation box, both air and resin may enter and exit, so it was set as an open pressure boundary, and the pressure was atmospheric pressure $P_{atm}$;
(3) Impregnation box wall: the fluid velocity perpendicular to the inner wall of the impregnation box was 0, and the wall was a no-slip wall.

## 4. Results and Discussion

### 4.1. Resin Flow and Impregnation Time in Impregnation Box

**Figure 5:**
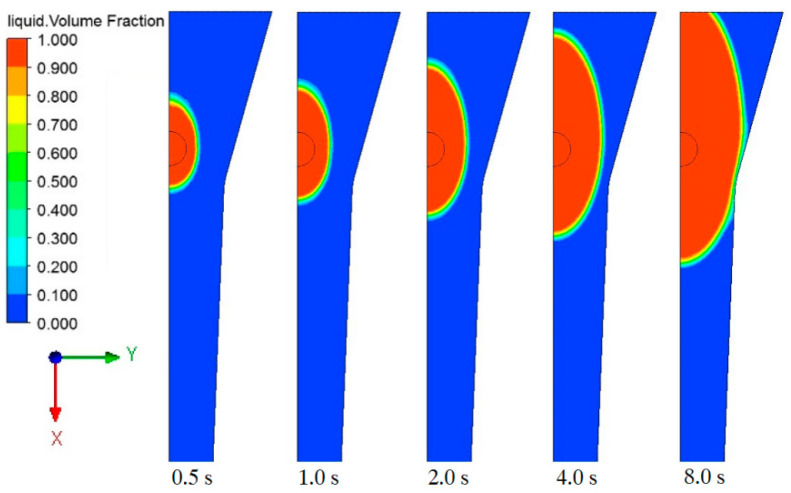
Resin flow distribution in the impregnation box at different times.

The complete impregnation of the resin to the fiber reinforcement in the injection box was the premise of preparing pultruded products with qualified performance. The viscosity of the resin used in this work was as low as 50 mPa·s at 100 °C and can maintain more than 25 min [27], which fully met the residence time requirements of the resin in the impregnation box. Therefore, the injection pressure (*P_i_*) was set to 0.5 bar, and pulling rate was set to 0 cm/min preliminarily to explore the resin flow. The cross section of calculation domain *z* = 0 is intercepted, and the flow distribution state of resin is shown in Figure 5.

It can be obtained from figure that the penetration rate along the *x*-axis direction (fiber axial direction) was obviously faster than that along the *y*-axis direction (fiber radial direction). At the same time, the permeation rate of the resin reflux in the negative direction of the *x*-axis was faster than that in the positive direction of the *x*-axis. This was because the permeability resistance along the *y*-axis was large, as shown in Figure 2b. In the positive *x*-axis direction, due to the compression of the cross-section size, the fiber volume fraction gradually increased, the porosity decreased, and the permeability resistance increased greatly. This can also be illustrated from the variation of porosity along the *x*-axis in Figure 2c.

When *t* = 8.0 s, the resin had overflowed from the entrance of the impregnation box, which would lead to material waste or oxidative deactivation of the reaction mixture. In the process of injection pultrusion, the fiber would move forward along the *x*-axis under the action of the puller. When the pulling speed matched the resin injection flow, the resin reflux can be effectively controlled, but an excessively high pulling rate would shorten the residence time of the reinforcement in the impregnation box, so even the fiber bundle cannot be fully penetrated. Therefore, optimization of process parameters and effective control of resin reflow were also the aims of this work.

**Figure 6:**
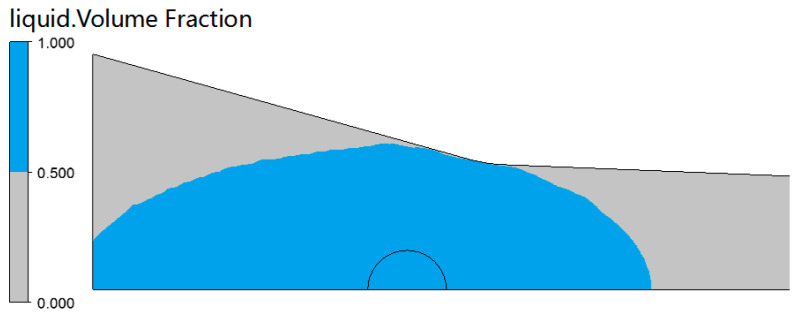
Flow front distribution of resin at *t* = 5.60 s.

Since the impregnation box had only one injection port, the time for the resin to completely penetrate the fiber bundle was the time for the resin to contact the wall of the impregnation box. After that, the complete filling of the resin can be ensured under the compression structure of the box cavity. When *t* = 5.60 s, the resin flow front (*α_r_* = 0.5) reaches the wall of the impregnation box, and the resin flow state distribution is shown in Figure 6.

### 4.2. Effect of the Pulling Rate

**Figure 7:**
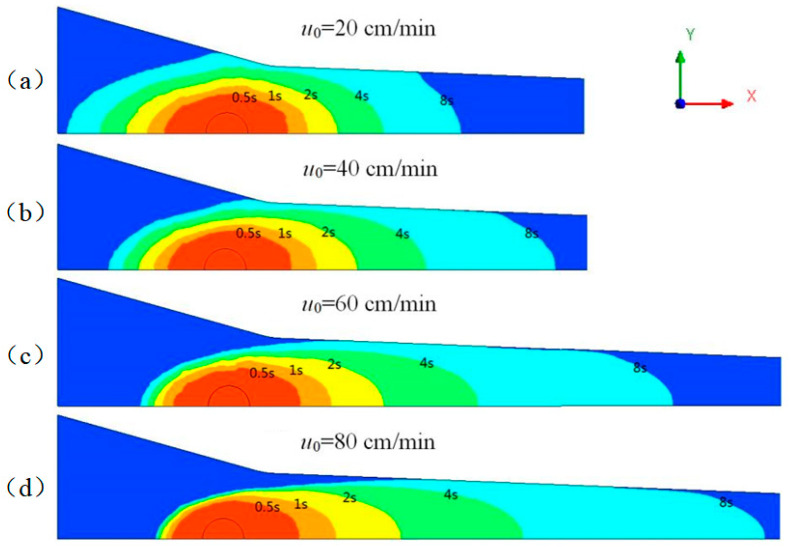
The resin flow front with time under different pulling rates: (**a**) *u*_0_ = 20 cm/min; (**b**) *u*_0_ = 40 cm/min; (**c**) *u*_0_ = 60 cm/min; (**d**) *u*_0_ = 80 cm/min.

Pulling rate was not only an important but also the most easily modified parameter in the pultrusion process [32]. Low pulling rate made it easier to control the forming of composite materials, but it would reduce production efficiency. Although productivity was improved at high pulling rate, the impregnation of fiber bundles was difficult to guarantee. It was necessary to explore a suitable range of pulling rate. The effect of different pulling rates on resin flow was studied when the injection pressure was maintained at 0.5 bar. The distribution of the resin flow at different moments of the *z* = 0 section is shown in Figure 7.

As can be seen from the figure, the ultra-low viscosity resin had strong permeability and could fully impregnate the fiber bundle within 8 s. The pulling rate and impregnation time were not completely positively correlated, and the relationship between them is shown in Table 4. In the range of pulling rate 0–20 cm/min, due to the high permeability along the radial direction of the fiber in Region 1, the resin penetrated rapidly, thus shortening the impregnation time. When the pulling rate was further increased, the penetration rate of the resin along the positive *x*-axis would increase, but the permeability along the radial direction of the fiber would be significantly reduced due to the compression of the box structure, resulting in a severe drop in the resin flow, which required a longer impregnation time. 

**Figure 8:**
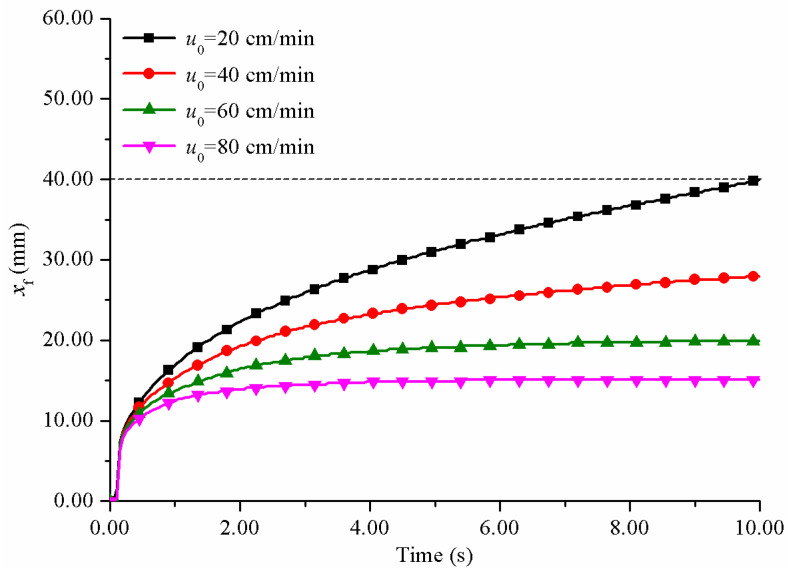
The relationship between resin reflux distance and time at different pulling rates.

It can also be seen that it was difficult to control the resin reflux at low pultrusion speed. When the pultrusion speed was 20 cm/min, the resin had almost overflowed to the inlet of the impregnation box at 8 s. Pultrusion cannot run stably for long time under this condition. The resin reflux phenomenon was most serious on the line of *y* = 0. The relationship between the reflux distance (*x*_f_) and time under different pulling rates on *y* = 0 line is studied, as shown in Figure 8. When *x*_f_ reached 40 mm, it meant that the resin would overflow from the inlet of the impregnation box.

Increasing the pulling rate can significantly reduce the reflux distance of resin. When the pulling rate exceeded 60 cm/min, the resin reflux distance increased to a certain value and remained basically unchanged. This was because increasing the pulling rate made the fiber movement and resin reflux reached a dynamic balance so as to maintain the resin flow in a stable state. The resin reflux was effectively controlled. Further increasing the pulling rate would shorten the reflux distance of resin. When the pulling rate was 80 cm/min, the resin reflux distance was only 15.1 mm. It did not mean that the faster the pulling rate, the better. However, when the pulling rate exceeded a certain range, the resin moved too fast along the positive direction of the *x*-axis, resulting in the resin flowing out of the impregnation box before contacting the wall, resulting in defects at the corners of the composite products.

### 4.3. Effect of the Injection Pressure

**Figure 9:**
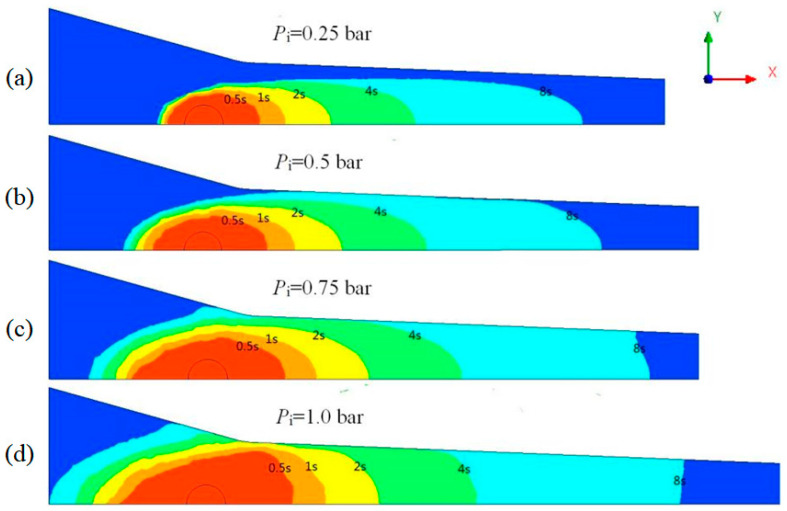
Flow front distribution of resin at different time with different injection pressures: (**a**) *P*_i_ = 0.25 bar; (**b**) *P*_i_ = 0.5 bar; (**c**) *P*_i_ = 0.75 bar; (**d**) *P*_i_ = 1.0 bar.

Injection pressure was another key process parameter during injection pultrusion process. Figure 9 shows the comparison of resin flow with time at various injection pressures in the section *z* = 0 of the box when the pulling rate was 60 cm/min.

It can be seen from the figure that when the injection pressure was 0.25 bar, the resin failed to flow to the wall of the impregnation box within 8.0 s. With the increase in injection pressure, the resin permeation rate along the fiber radial direction gradually increased and the impregnation time was greatly shortened. The impregnation time under different injection pressures is shown in Table 5.

**Figure 10:**
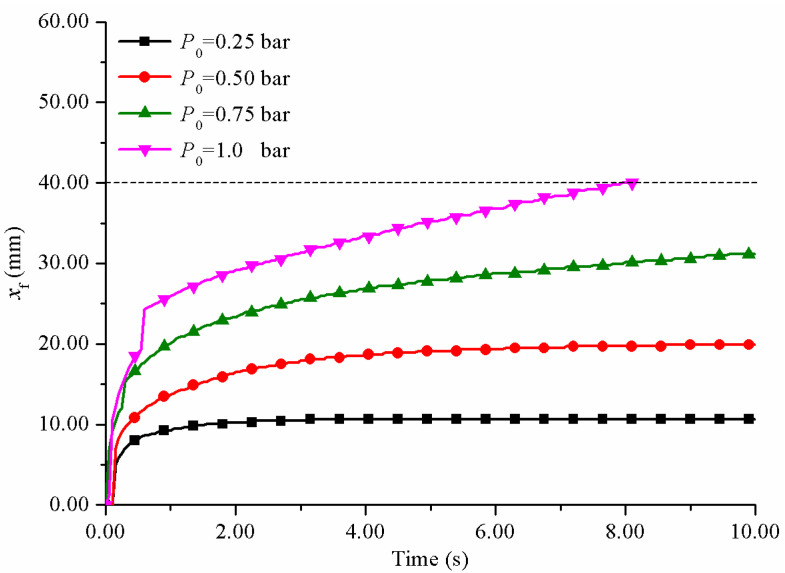
The relationship between resin reflux distance and time at different injection pressures.

With the increase in injection pressure, the reflux distance along the negative direction of *x*-axis also increased, resulting in more serious resin reflux. The relationship between resin reflux distance and time on the line *y* = 0 at different injection pressures is shown in Figure 10.

The lower the injection pressure, the shorter the reflux distance and the easier it was to control the resin reflux. When the injection pressure was 0.25 bar, the resin reflux distance was only 10.7 mm, which was effectively controlled. When the injection pressure was 0.75 bar or more, the reflux distance was difficult to be controlled within 40 mm. A faster pulling rate was required to match the high injection pressure. Although providing a higher injection pressure can improve production efficiency, the high injection pressure was not recommended. In this study, the porous media model was used to simulate the resistance of fiber reinforcement to resin flow. In fact, the fiber reinforcement was flexible. Under high injection pressure, the resin flow would impact the fiber reinforcement, resulting in fiber compression, resulting in the defect of uneven fiber dispersion in the product [33], which seriously affected the performance of the product. Therefore, the injection pressure should be as low as possible when the impregnation requirements were met. It was more appropriate to set the injection pressure in the range of 0.25–0.5 bar.

## 5. Conclusions

In this work, modeling and numerical simulation of impregnation in reactive injection pultrusion of GF/PA6 composites were carried out. The process within the impregnation box was regarded as a gas–liquid two-phase seepage flow in a moving porous medium. Considering the influence of air in the reinforcing fibers, the impregnation process was simulated and analyzed by ANSYS CFX 15.0 software. The following conclusions can be obtained:

(1) The caprolactam resin had great permeability; even the injection pressure was only 0.5 bar, and the complete penetration of reinforced fiber can be realized in 0.56 s;
(2) When the injection pressure was 0.5 bar, increasing the pulling rate could shorten the impregnation time in the range of 0–20 cm/min; when the pulling rate was further increased, the impregnation time would increase; increasing the pulling rate can significantly control the reflux distance of resin in the impregnation box. The reflux distance of resin can be controlled to 15.1 mm when the pulling rate was increased to 80 cm/min;
(3) Increasing the injection pressure can greatly shorten the resin impregnation time, but it would significantly increase the resin reflux distance and cause the resin to overflow from the entrance of the impregnation box; a high pulling rate was required to match the high injection pressure; it was more appropriate to set the injection pressure in the range of 0.25–0.5 bar when the impregnation requirements were met.

## Figures and Tables

**Table 1:** Geometric parameters of impregnation box.

Geometric Parameters	Value
Total length of impregnation box (*L*)	200 mm
Height (*W*)	4 mm
Region 1 length (*L*_1_)	50 mm
Region 2 length (*L*_2_)	150 mm
Entrance width (*H*_i_)	60 mm
Region junction width (*H*_m_)	32 mm
Exit width (*H*_o_)	20 mm
Length from injection center to Entrance (*X*)	40 mm
Region 1 cone angle (tan*σ*)	0.28
Region 2 cone angle (tan*η*)	0.05

**Table 2:** Physical parameters of air and liquid resin.

Materials	Density (kg/m^3^)	Viscosity (mPa·s)	Surface Tension Coefficient (N/m)	Contact Angle
Resin (liquid)	950	50	0.034	34°
Air (gas)	0.946	0.0218	—	—

**Table 3:** Permeability model parameters.

Fiber Arrangement	*D*_f_	*C*_1_	*V*_fmax_	*c*
Hexagonal Arrangement	17 μm	0.231	0.907	53

**Table 4:** The relationship between pultrusion speed and impregnation time.

Pulling Rate (cm/min)	0	20	40	60	80
Impregnation Time (s)	5.60	5.15	6.25	6.90	7.20

**Table 5:** The relationship between injection pressure and impregnation time.

Injection Pressure (bar)	0.25	0.50	0.75	1.00
Impregnation Time (s)	>8.0	5.60	4.05	2.60

## Data Availability

The data presented in this study are available on request from the corresponding author.
